# Dataset on the evaluation of an ELISA assay using Yersinia pseudotuberculosis O-antigen for serodiagnosis in comparison with agglutination antibody test

**DOI:** 10.1016/j.dib.2026.113122

**Published:** 2026-08-03

**Authors:** Hidemasa Izumiya, Hiroshi Nakajima, Yuta Kawakami, Jun Kawase, Yukihiro Akeda

**Affiliations:** a Department of Bacteriology I, National Institute of Infectious Diseases, Japan Institute for Health Security, Japan; b Okayama Prefectural Institute for Environmental Science and Public Health, Japan; c Division of Bacteriology, Shimane Prefectural Institute of Public Health and Environmental Science, Japan

**Keywords:** *Yersinia pseudotuberculosis*, O-antigen, ELISA, Agglutination, Serodiagnosis

## Abstract

*Yersinia pseudotuberculosis* is an enteric pathogen that causes a variety of symptoms as well as enterocolitis and has been implicated as a potential infectious trigger of Kawasaki disease. The pathogen exhibits complex epidemiological and immunological characteristics that vary by region and host species. Conventional diagnostic methods such as culture and agglutination antibody assay with O-antigens have limitations in sensitivity, specificity, and interpretability. To address these issues, enzyme-linked immunosorbent assays (ELISAs) using *Y. pseudotuberculosis* O-antigens have been developed to enable quantitative serodiagnosis. This study provides a dataset for evaluating an indirect ELISA using purified *Y. pseudotuberculosis* O-antigens. A total of 49 archived serum samples previously collected from suspected *Y. pseudotuberculosis* cases were analysed. Fifteen O-antigens representing major serogroups were evaluated, and ELISA results were compared with traditional agglutination antibody assay results. Diagnostic performance was assessed using receiver operating characteristic (ROC) analysis, including the calculation of sensitivity and specificity. Cutoffs were also determined using the mean + 2 standard deviation approach. The dataset demonstrates antigen-dependent ELISA reactivity and the diagnostic performance. Antigen-specific ROC analyses and performance metrics are provided for all tested O-antigens, together with an additional analysis of the composite analytical variable (O2abc). These data could facilitate the development, optimization, and standardization of O-antigen-based serological assays for the diagnosis of *Y. pseudotuberculosis* infection.

Specifications Table

**Table:** 

Subject	Biology
Specific subject area	Diagnostic microbiology; bacterial zoonoses; serology
Type of data	Figures, tables, analysed data
Data collection	Indirect ELISA using purified *Y. pseudotuberculosis* O-antigen; absorbance measured at 450 nm with microplate reader
Data source location	Okayama, Japan
Data accessibility	Data available upon publication via Mendeley Data repository Izumiya, Hidemasa (2026), “ELISA and agglutination test for the serodiagnosis of *Y*ersinia *pseudotuberculosis* infection”, Mendeley Data, V1, doi:10.17632/bwdpmxy4nx.1 https://data.mendeley.com/datasets/bwdpmxy4nx/1
Related research article	Nakajima et al. [1]

## 1 Value of the Data

• These data provide validated ELISA performance metrics using *Y. pseudotuberculosis* O-antigen, benchmarked against traditional agglutination antibody test.
• The dataset allows evaluation of concordance between ELISA and agglutination results, offering a practical framework for serological standardization.
• The dataset may support improved differential diagnosis and understanding of Kawasaki Disease-like syndromes associated with *Yersinia* infection.

## 2 Background

*Y. pseudotuberculosis* is a facultatively anaerobic Gram-negative rod belonging to genus *Yersinia* and is closely related to *Y. enterocolitica* [2]. *Y. pseudotuberculosis* infection presents a variety of syndromes such as fever, rash, desquamation, strawberry tongue, lymphadenopathy, and conjunctivitis, and has also been associated with systemic inflammatory responses like Kawasaki disease [3,4]. Epidemiological studies have demonstrated the presence of *Y. pseudotuberculosis* in humans, animals, and food sources, indicating zoonotic transmission [5,6]. *Y. pseudotuberculosis* consists of 21 types in O-antigen based serotyping, of which serotypes O:1a, O:1b, O:2a, O:2b, O:2c, O:3, O:4b, O:5a, O:5b, O:6, O:10, and O:15 are known to be associated to human infections [3,7,8]. Although culture remains the diagnostic gold standard, it is often time-consuming and insensitive, particularly after antibiotic treatment [9]. Serological methods, including agglutination antibody tests, provide supportive diagnostic information but are prone to cross-reactivity and subjective interpretation [1]. Therefore, quantitative ELISA methods are increasingly applied to improve diagnostic precision [10].

The objective of this dataset is to evaluate the diagnostic utility of an ELISA based on *Y. pseudotuberculosis* O-antigen by comparing its results with conventional O-antigen agglutination antibody tests. The study also explores potential serological overlap with Kawasaki disease.

## 3 Data Description

The dataset is in an Excel file. It consists of 735 ELISA OD values of a total of 49 serum samples tested against 15 types of O-antigens (O:1a, O:1b, O:1c, O:2a, O:2b, O:2c, O:3, O:4a, O:4b, O:5a, O:5b, O:6, O:7, O:10, and O:15). The corresponding O-antigen agglutination antibody tests are also included.

**Fig. 1:**
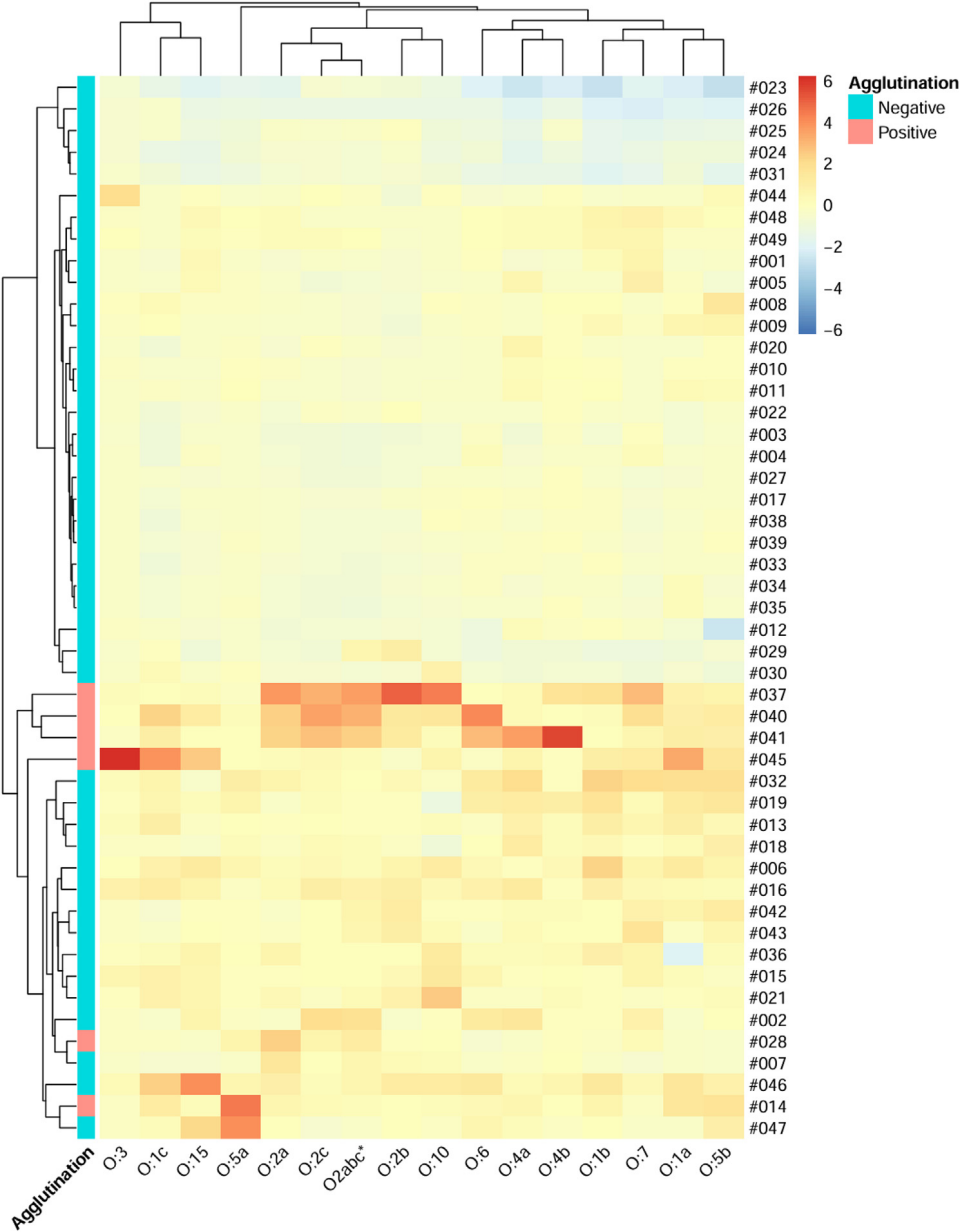
Heatmap of ELISA reactivity against 15 *Yersinia pseudotuberculosis* O-antigens. Heatmap, created using pheatmap package of R, showing Z-score-transformed ELISA optical density (OD) values obtained using 15 *Y. pseudotuberculosis* O-antigens and 49 serum samples. Rows represent serum samples and columns represent O-antigens. For each antigen, OD values were standardized to Z-scores to facilitate comparison of reactivity patterns across antigens with different signal distributions. Positive Z-scores indicate higher-than-average reactivity, whereas negative Z-scores indicate lower-than-average reactivity. Row annotations indicate agglutination test results (positive or negative). *, O2abc represents a composite analytical variable, calculated as the maximum ELISA OD value among O:2a, O:2b, and O:2c for each serum sample, and does not represent an additional biological O-antigen.

The heatmap (Fig. 1) visualizes antigen-specific antibody response patterns and potential cross-reactivity among O-antigens. To enable comparison among antigens with different baseline OD distributions, ELISA values were standardized to Z-scores prior to heatmap visualization.

Table 1 summarizes antigen-specific diagnostic performance estimated using receiver operating characteristic (ROC) analysis and cutoff values determined by Youden’s index. For each antigen, the numbers of agglutination-positive and agglutination-negative sera, area under the ROC curve (AUC), optimal cutoff value, confusion matrix counts including true positive (TP), true negative (TN), false positive (FP), and false negative (FN), sensitivity, specificity, positive predictive value (PPV), negative predictive value (NPV), and overall accuracy are provided. Wilcoxon rank-sum test p-values, Student’s t-test p-values, and Cohen’s d effect sizes were calculated to assess differences in ELISA reactivity between agglutination-positive and agglutination-negative sera.

**Table 1:** Performance of ELISAs using individual O-antigens and the O2abc composite analytical variable based on ROC analysis and Youden’s index.

Antigen	Positive	Negative	AUC	AUC_95%CI	Cutoff	TP	TN	FP	FN	Sensitivity	Specificity	PPV	NPV	Accuracy	Wilcoxon_p*	Ttest_p*	Cohens_d
O:1c	1	48	0.656	NA	0.070	1	31	17	0	1.000	0.646	0.056	1.000	0.653	0.621	NA	NA
O:2a	2	47	0.989	0.960–1.000	0.285	2	46	1	0	1.000	0.979	0.667	1.000	0.980	0.021	0.124	−4.103
O:2b	1	48	1.000	NA	0.442	1	48	0	0	1.000	1.000	1.000	1.000	1.000	0.097	NA	NA
O:2c	3	46	1.000	1.000–1.000	0.421	3	46	0	0	1.000	1.000	1.000	1.000	1.000	0.004	0.002	−5.802
O:3	1	48	1.000	NA	0.852	1	48	0	0	1.000	1.000	1.000	1.000	1.000	0.097	NA	NA
O:4a	1	48	1.000	NA	0.176	1	48	0	0	1.000	1.000	1.000	1.000	1.000	0.097	NA	NA
O2abc	4	45	0.994	0.979–1.000	0.265	4	44	1	0	1.000	0.978	0.800	1.000	0.980	0.001	0.013	−4.445

*p-value. NA, not applicable because statistical estimation could not be performed due to an insufficient number of agglutination-positive sera.

**Fig. 2:**
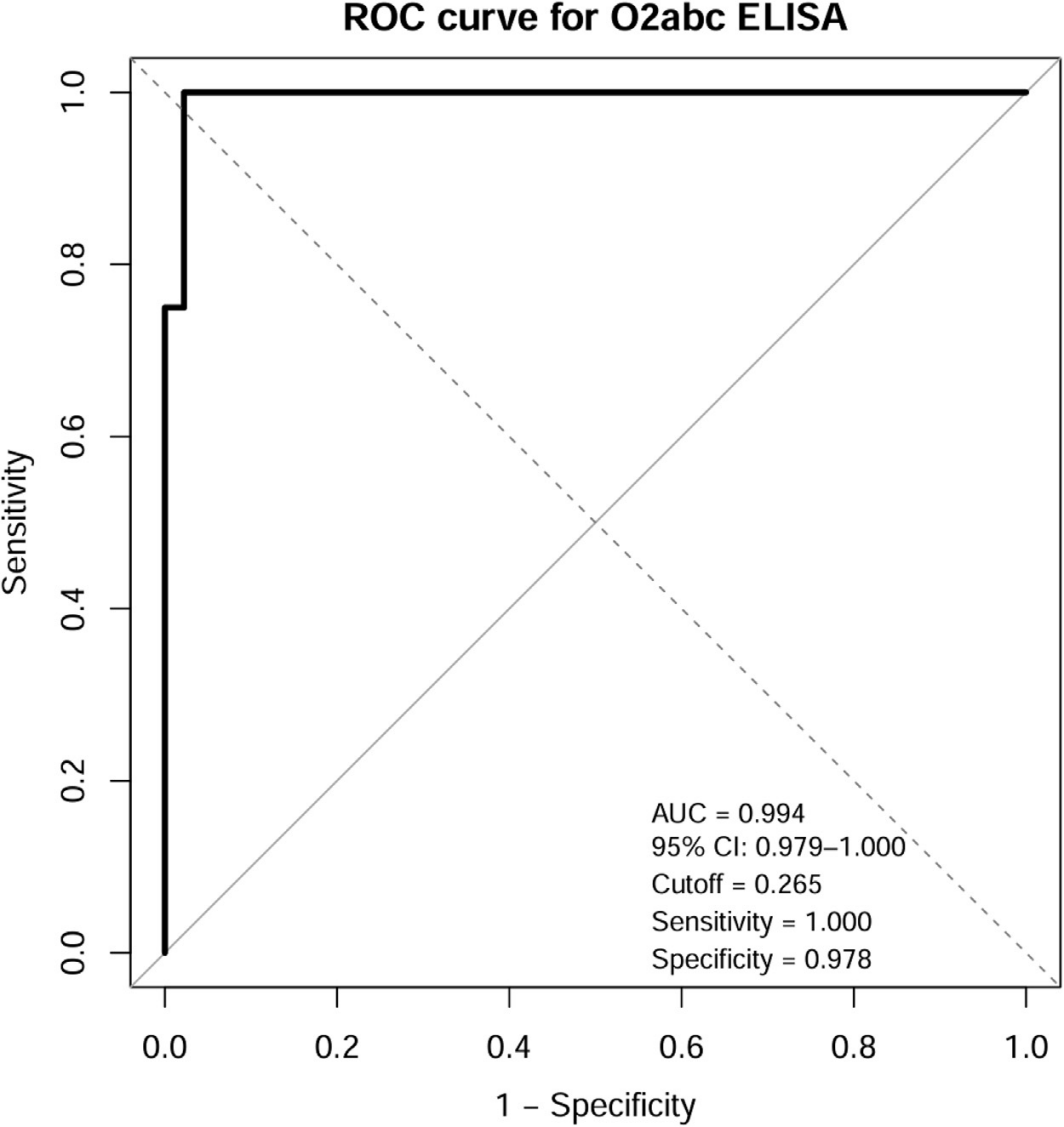
Receiver operating characteristic (ROC) curve for the O2abc ELISA. ROC curve was created by pROC package of R. O2abc represents a composite analytical variable, calculated as the maximum ELISA OD value among O:2a, O:2b, and O:2c for each serum sample. Agglutination assay results were used as the reference method for ROC analysis. The area under the curve (AUC) was 0.994 (95% confidence interval [CI]: 0.979–1.000), indicating excellent discrimination between agglutination-positive and agglutination-negative sera. The optimal cutoff value determined using Youden’s index was 0.265, corresponding to a sensitivity of 1.000 and a specificity of 0.978. The x-axis represents the false positive rate (1 - specificity), while the y-axis represents the true positive rate (sensitivity).

Antigen-specific ROC analyses were performed to evaluate diagnostic performance and determine optimal cut-off values for each O-antigen. In addition, a composite analytical variable, O2abc, was designated. O2abc was defined as the maximum ELISA optical density (OD) value obtained using O:2a, O:2b, and O:2c antigens. This included more agglutination-positive sera (n = 4) than most other O-antigens and showed the highest overall diagnostic performance. This variable may improve the serological discrimination of those infected with O:2-related strains than analyses based on individual O:2 antigens alone. Therefore, the reactivity of O2abc was included in Fig. 1, and the ROC curve for O2abc is presented as a representative example in Fig. 2, whereas the complete diagnostic performance results for all evaluated antigens are summarized in Table 1.

A two-way ANOVA demonstrated significant effects of O-antigen type, agglutination status, and their interaction on ELISA OD values (all p < 0.001), indicating that the relationship between ELISA reactivity and agglutination results varied among O-antigens.

Table 2 presents corresponding diagnostic performance estimates obtained using the conventional mean + 2 standard deviation (SD) cutoff approach based on agglutination-negative sera. The table includes the mean and SD of negative sera, calculated cutoff values, confusion matrix counts, and diagnostic performance metrics. These complementary analyses enable comparison between a commonly used empirical cutoff method and ROC-based optimization.

**Table 2:** Performance of ELISAs using cutoff values defined by the mean + 2SD method.

Antigen	Mean_negative	SD_negative	Cutoff_mean2SD	TP	TN	FP	FN	Sensitivity	Specificity	PPV	NPV	Accuracy
O:1c	0.066	0.065	0.195	0	45	3	1	0.000	0.938	0.000	0.978	0.918
O:2a	0.055	0.072	0.199	2	44	3	0	1.000	0.936	0.400	1.000	0.939
O:2b	0.073	0.075	0.222	1	46	2	0	1.000	0.958	0.333	1.000	0.959
O:2c	0.080	0.076	0.233	3	44	2	0	1.000	0.957	0.600	1.000	0.959
O:3	0.051	0.080	0.211	1	46	2	0	1.000	0.958	0.333	1.000	0.959
O:4a	0.027	0.043	0.114	1	47	1	0	1.000	0.979	0.500	1.000	0.980
O2abc	0.097	0.083	0.263	4	44	1	0	1.000	0.978	0.800	1.000	0.980

For antigens O:1a, O:1b, O:4b, O:5a, O:5b, O:6, O:7, O:10 and O:15, no agglutination-positive sera were available. Therefore, ROC analysis could not be performed, and cut-off values were provisionally defined using the mean + 2SD approach. Supplementary Table S1 summarizes the mean OD values, SDs, and resulting cut-off values for these antigens.

## 4 Experimental Design, Materials and Methods

### 4.1 Human serum samples

Human sera used in this study were obtained from a previous research project [1] (https://www.jstage.jst.go.jp/article/kansenshogakuzasshi/96/1/96_8/_article/-char/ja/). The sera were collected from suspected *Y. pseudotuberculosis* cases, including those diagnosed with Kawasaki disease. Detailed clinical information was unavailable because the samples had been anonymized. The present analysis was performed retrospectively using archived residual sera remaining after completion of the original study. Samples were included when sufficient serum volume was available for ELISA testing. Detailed information regarding patient recruitment, specimen collection, and agglutination testing has been described previously [1]. A total of 49 serum samples from 29 individuals were used in this study. Sera were heat-inactivated (56 °C for 30 min) before testing.

### 4.2 Tube agglutination assay

Agglutination antibody titers were determined using the standard tube agglutination method [1]. Briefly, two-fold serial dilutions of sera were mixed with heat-killed whole-cell bacterial antigens. After incubation at 50 °C for 2 h followed by overnight standing at room temperature, the highest dilution showing visible agglutination was recorded as the antibody titre. An agglutination titer of ≥1:160 was regarded as positive.

### 4.3 O-antigen preparation for ELISA

O-antigens used in this study were prepared in-house. Strains of *Y. pseudotuberculosis* used in this study were derived from a previous study [1]. Bacterial strains were cultured on Tryptic Soy agar (Becton Dickinson and Company, NJ, USA) at 30 °C for 48 h. O-antigens (lipopolysaccharide) were purified using a modified hot phenol-water extraction method [11,12]. Purified *Y. pseudotuberculosis* O-antigens were quantified and standardized based on endotoxin activity measured using a Limulus Amebocyte Lysate assay (LAL Chromogenic Endotoxin Quantitation Kit; Thermo Fisher Scientific, MI, USA) and used as the coating antigen for an indirect ELISA. The O-antigens used in this study were O:1a, O:1b, O:1c, O:2a, O:2b, O:2c, O:3, O:4a, O:4b, O:5a, O:5b, O:6, O:7, O:10, and O:15. Antigenicity of O:1a, O:1b, O:1c, O:2a, O:2b, O:2c, O:3, O:4a, O:4b, O:5a, O:5b, and O:6 antigens was confirmed using O-group-specific antisera (Denka, Japan).

### 4.4 ELISA procedure

ELISAs were performed using MaxiSorp 96-well microplates (Thermo Scientific Clear Flat-Bottom Immuno Nonsterile 96-Well Plates with MaxiSorp; Thermo Fisher Scientific). Microtiter plates were coated with 100 ng/well of O-antigen using a coating buffer (Carbonate-Bicarbonate Buffer; Sigma-Aldrich, MO, USA) at 37 °C for 2 h in duplicate, blocked with Pierce™ Protein-Free (PBS) Blocking Buffer (Thermo Fisher Scientific) according to the manufacturer’s instructions, and incubated with 1:320-diluted human sera for 2 h. 1:4000-diluted HRP-conjugated goat anti-human IgM antibodies (Thermo Fisher Scientific) and TMB substrate (1-Step Turbo TMB-ELISA Substrate Soution; Thermo Fisher Scientific) were used for detection, and absorbance was read at 450 nm. Duplicate OD values were averaged after sequential subtraction of the corresponding blank well (without serum) and uncoated well (without O-antigen) values to correct for background absorbance and non-specific serum binding. No standardized positive or negative control sera were included because this study was designed as a retrospective evaluation using archived sera.

### 4.5 Data analysis

Diagnostic performance was evaluated using the tube agglutination assay as the reference method. ROC curve analysis was performed, and AUC with 95% confidence intervals was calculated. Optimal cutoff values were determined using Youden’s index. In addition, a conventional cutoff value was calculated for each antigen as the mean OD of agglutination-negative sera plus two standard deviations (mean + 2SD). Diagnostic performance metrics, including sensitivity, specificity, PPV, NPV, accuracy, and confusion matrix counts (TP, TN, FP, and FN), were calculated for both cutoff approaches. Differences in ELISA OD values between agglutination-positive and agglutination-negative sera were assessed using both Student’s *t*-test and the Wilcoxon rank-sum test. Effect sizes were estimated using Cohen’s d. Heatmap visualization and statistical analyses were performed in R (version 4.1.2).

All diagnostic performance analyses were performed separately for each O-antigen. In addition, a composite analytical variable (O2abc) was calculated as the maximum OD value among O:2a, O:2b, and O:2c antigens and was evaluated separately.

## Limitations

The primary limitation of this dataset lies in the limited sample size due to the rarity of the disease. Because the study used archived sera selected on the basis of sample availability, the dataset represents a convenience sample rather than a prospectively designed diagnostic validation cohort. Furthermore, the number of agglutination-positive sera available for some serotypes was limited, which may have affected the precision of performance estimates. The agglutination assay was used as the reference method; however, the possibility of non-specific agglutination and discrepancies between agglutination and ELISA-based measurements cannot be excluded. Additionally, antibodies against *Yersinia* outer proteins (Yops), which are also commonly used as serological targets in *Yersinia* infections, were not evaluated in the present study. Therefore, the relationship between O-antigen reactivity and Yop-specific antibody responses was not assessed. The study population was regionally constrained, and further validation across different geographical areas and outbreak settings is warranted. Cross-reactivity with other *Yersinia* species or enteric pathogens cannot be completely excluded and should be evaluated in future studies.

## Ethics Statement

All serum samples were anonymized prior to analysis. The study was approved by the institutional ethics committees of National Institute of Infectious Diseases (Approval No. 1306) and Okayama Prefectural Research Center of Environment and Public Health (Approval No. Kanpo-613), and written informed consent was obtained from all participants or their guardians.

## CRediT Author Statement

**Hidemasa Izumiya:** Conceptualization, Methodology, Formal analysis, Writing - Original Draft, Visualization. **Hiroshi Nakajima:** Conceptualization, Methodology, Resources, Writing - Review & Editing. **Yuta Kawakami, Jun Kawase:** Resources, Writing - Review & Editing. **Hiroyuki Akeda:** Writing - Review & Editing.

## Declaration of Generative AI and AI-Assisted Technologies in the Writing Process

During the preparation of this work, the authors used ChatGPT in order to improve the language and readability of the manuscript and to assist with English editing. After using this tool, the authors reviewed and edited the content as needed and take full responsibility for the content of the published article.

## Data Availability

Mendeley DataELISA and agglutination test for the serodiagnosis of Yersinia pseudotuberculosis infection (Original data)

## Appendix Supplementary materials

Image, application 1
