# Supplementary material for: Dataset on the evaluation of an ELISA assay using Yersinia pseudotuberculosis O-antigen for serodiagnosis in comparison with agglutination antibody test
Source: Data Brief. 2026 Aug 3;68:113122. doi: 10.1016/j.dib.2026.113122 (PMC13475374; doi:10.1016/j.dib.2026.113122)
Supplement: Supplementary file 1 [file mmc1.docx]

**Table S1. Mean OD values, standard deviations, and cut-off values (mean + 2 SD) for O-antigen serogroups for which no agglutination-positive sera were available.**

| Antigen | N | Mean OD | SD OD | Cutoff (Mean + 2SD) |
| --- | --- | --- | --- | --- |
| O1a | 49 | 0.028 | 0.043 | 0.114 |
| O1b | 49 | 0.022 | 0.039 | 0.100 |
| O4b | 49 | 0.012 | 0.052 | 0.116 |
| O5a | 49 | 0.031 | 0.072 | 0.175 |
| O5b | 49 | 0.024 | 0.044 | 0.112 |
| O6 | 49 | 0.028 | 0.058 | 0.144 |
| O7 | 49 | 0.035 | 0.046 | 0.126 |
| O10 | 49 | 0.046 | 0.075 | 0.195 |
| O15 | 49 | 0.037 | 0.057 | 0.152 |
